# Ureteral pseudodiverticulosis accompanied by urothelial carcinoma diagnosed by CT urography: a case report and review of the literature

**DOI:** 10.1259/bjrcr.20170111

**Published:** 2018-01-18

**Authors:** Hiroaki Sugiura, Hirotaka Akita, Eiji Kikuchi, Shuji Mikami, Nozomi Hayakawa, Keiichi Narita, Masahiro Jinzaki

**Affiliations:** 1 Department of Diagnostic Radiology, Keio University School of Medicine, Shinjuku-ku, Tokyo, Japan; 2 Department of Urology, Keio University School of Medicine, Shinjuku-ku, Tokyo, Japan; 3 Department of Diagnostic Pathology, Keio University School of Medicine, Shinjuku-ku, Tokyo, Japan; 4 Department of Urology, Nerima General Hospital, Nerima-ku, Tokyo, Japan

## Abstract

Ureteral pseudodiverticulosis is a relatively rare condition and has been diagnosed by retrograde urography and excretory urography. Ureteral pseudodiverticulosis is also suspected to be a potential risk factor for the development of urothelial carcinoma. We report the case of a male in his 70 s who was suspected to have right ureteral pseudodiverticulosis accompanied by multifocal urothelial carcinoma based on CT urography findings. After surgery, the pathological findings confirmed the presence of ureteral pseudodiverticulosis and multifocal urothelial carcinoma in his right ureter and bladder. To the best our knowledge, this is the first reported case of ureteral pseudodiverticulosis with concurrent urothelial carcinoma detected by CT urography. Since CT urography has replaced excretory urography as the first-line imaging test for investigating patients with high risk for upper tract urothelial carcinoma, it is important to recognize the characteristic findings of ureteral pseudodiverticulosis on CT urography.

## Clinical presentation

A male in his 70s complaining of gross haematuria underwent a CT examination at another hospital, disclosing a mass in his right middle ureter. As there was no indication of hydronephrosis on the CT findings and the urine cytology results were negative, the possibility of an extraureteral tumour was considered and he was referred for further medical workup. His serum creatinine level was 0.94 mg dl^−1^ and his estimated glomerular filtration rate was 60.8 ml/min/1.73 m^2^.

## Imaging findings

**Figure 1.**
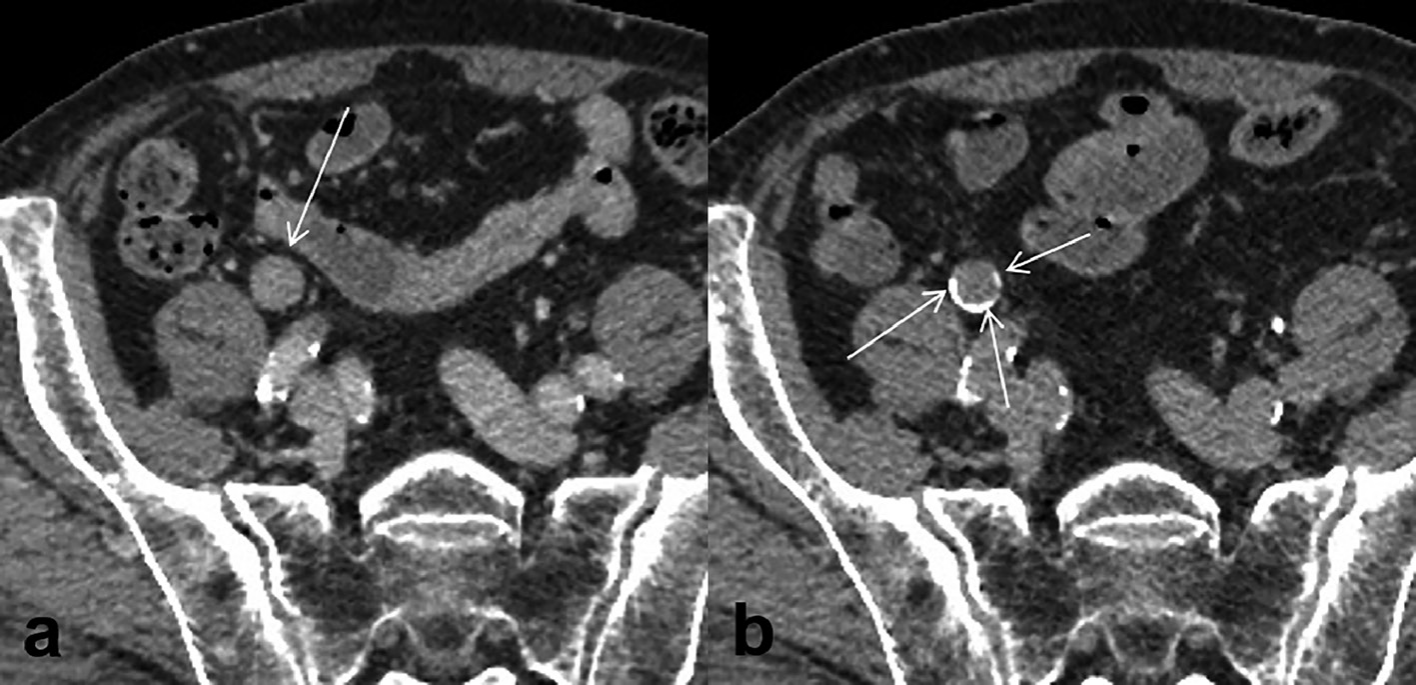
CT urography [(a) nephrographic phase; (b) excretory phase] A homogeneous mass (a: arrow) is visible in the right middle ureter during the nephrographic phase, and contrast medium retention (b: arrow) is seen around the mass during the excretory phase. Therefore, the mass can be interpreted as a papillary tumour growing within the ureteral lumen.

**Figure 2.**
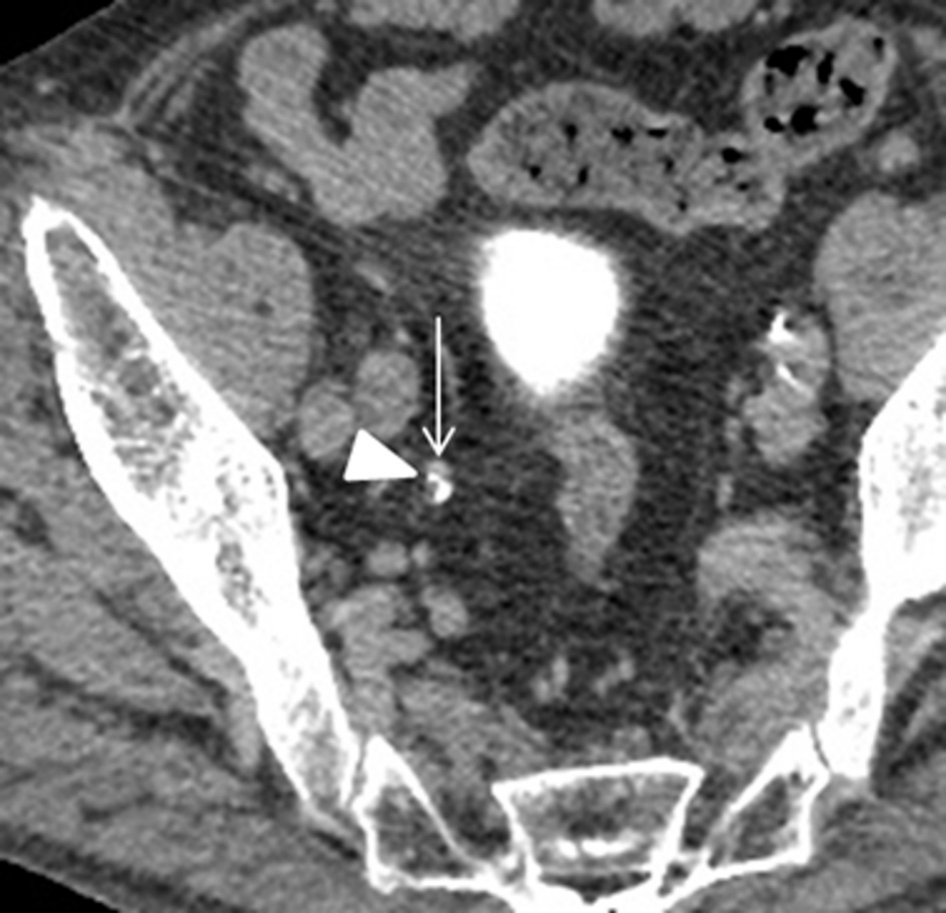
CT urography (excretory phase) During the excretory phase, a filling defect (arrow) of a few millimetres is visible in the right ureter distal to the lesion shown in Figure 1. A small ureteral cancer was suspected. A tiny outpouching of contrast medium (arrow head) possibly representing a diverticulum is visible around the tumour.

**Figure 3.**
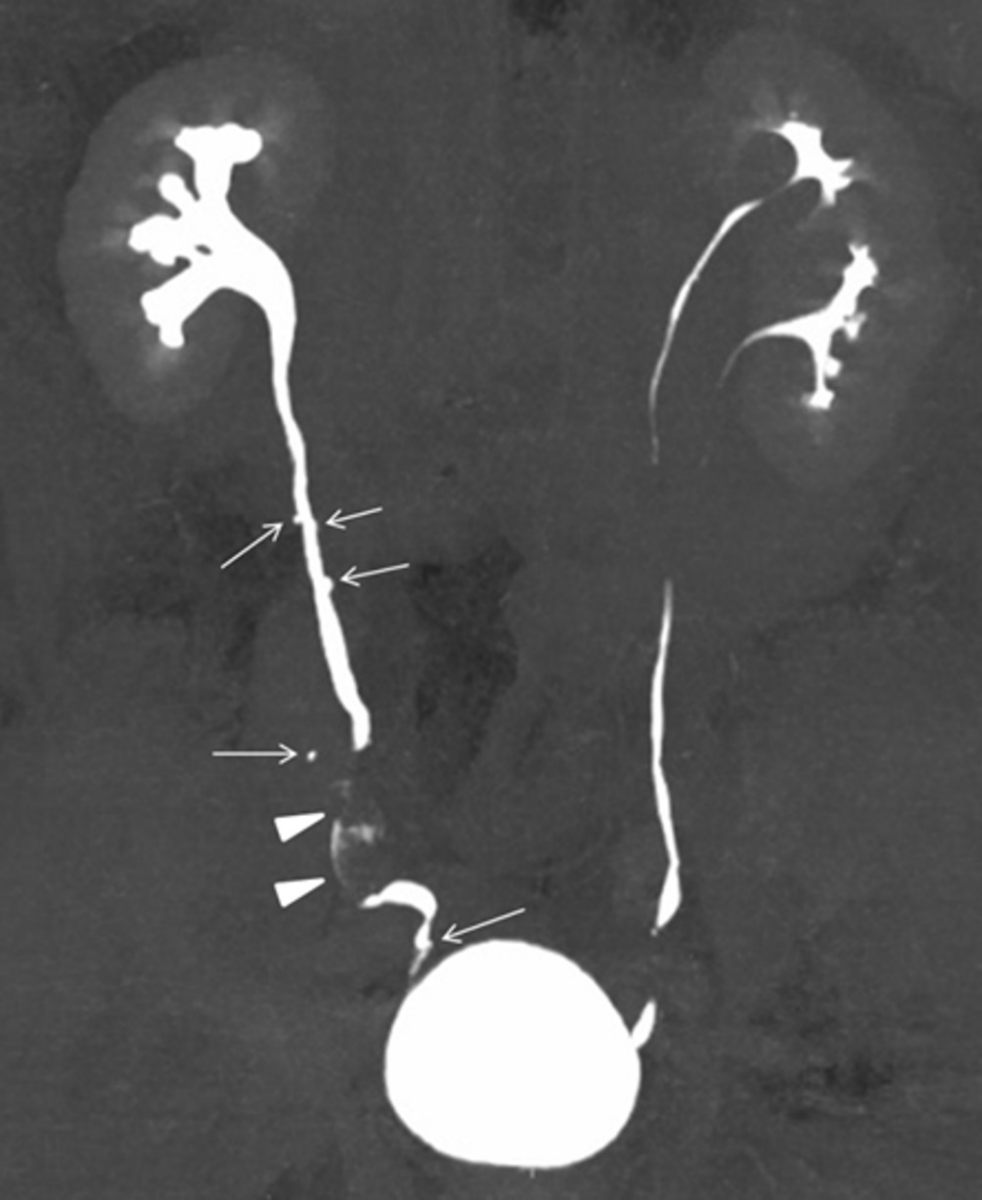
CT urography (excretory phase/maximum intensity projection image) While mild right hydronephrosis was present, the excretion of the contrast medium was satisfactory. Multiple outpouchings of contrast medium measuring a few millimetres (arrows) are visible in the right ureter, suggesting ureteral pseudodiverticulosis. The arrowhead indicates a filling defect due to the right middle ureteral cancer. Incomplete double renal pelvis and ureter on the left side are also visible.

At our hospital, CT urography revealed a 4.5 × 2 × 1.5 cm mass in the right middle ureter during the nephrographic phase (Figure 1a). During the excretory phase, the contrast medium was distributed around the rim of the mass (Figure 1b), which was visualized as an intraureteral papillary tumour. The CT attenuation values of the mass were 38 HU, 88 HU and 59 HU for the unenhanced, nephrographic and excretory phase, respectively. The presence of multiple tumours, each measuring a few millimetres in size, in the right distal ureter (Figure 2) and bladder was also suspected. Furthermore, multiple outpouchings of contrast medium of a few millimetres were also visualized in the right ureter during the excretory phase, presenting diverticular features (Figures 2 and 3). Based on these findings, the case was diagnosed as having pseudodiverticulosis of the right ureter with concurrent multifocal urothelial carcinoma in the right ureter and bladder.

## Treatment/outcome/follow-up

**Figure 4.**
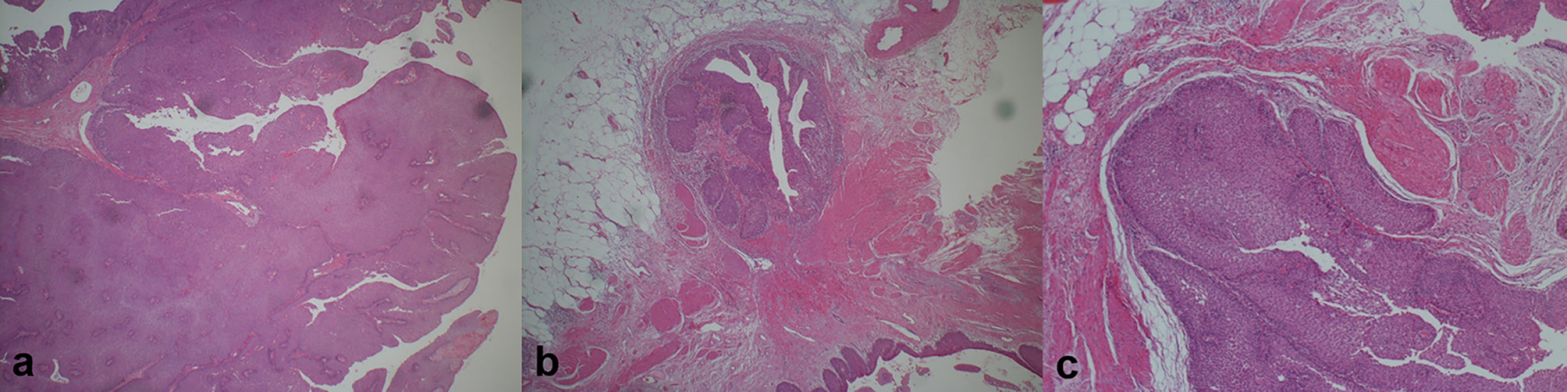
Histopathological photomicrographs with haematoxylin and eosin staining In the right middle ureter, a tumour with papillary growth and displaying pronounced stratification of a low-grade atypical urothelium is visible (a). The tumour also exhibits inverted growth, with the formation of cancer cell nests in the surrounding adipose tissue beyond the muscular layer; the lesion was diagnosed as pT3 (b). Distally, a 0.3 cm tumour in the right ureter was noted (c). The low-grade atypical urothelium shows a well-demarcated inverted growth even into the muscular layer, suggesting invasion into the diverticulum. The lesion was consequently diagnosed as pT2.

A transurethral resection of the bladder tumour was performed, and the vesical lesion was diagnosed as a non-invasive papillary urothelial carcinoma, low grade (pTa). Subsequently, a laparoscopic radical nephroureterectomy was performed after scrutiny with retrograde urography (ureteral washing cytology: positive) for the right ureteral tumours. The ureteroscopic evaluation before the nephroureterectomy was technically unsuccessful owing to tight adhesion of the main tumour. Histopathological examination revealed hyperplastic changes of the ureteral epithelium accompanied by fibrosis and irregular spreading of epithelial cells into the subepithelial connective tissue, indicating ureteral pseudodiverticulosis. Also noted were two invasive papillary urothelial carcinomas, low grade in the right ureter, one measuring 2 × 1.5 cm and the other measuring 0.3 cm located distally to the former (Figure 4). As cancer cell nests were evident even in a region beyond the muscular layer in the proximal larger tumour lesion, the lesion was diagnosed as pT3 (Figure 4b). In the distal smaller tumour lesion, the inverted proliferation of atypical urothelium even into the muscular layer was observed, suggesting invasion into the diverticulum (Figure 4c). The lesions were diagnosed as pT2.

On the 17th day post-radical nephroureterectomy, the patient was discharged without complications. At 1 year after the initial transurethral resection, multiple recurrent tumours were found in the bladder on cystoscopic follow-up. The patient underwent a second transurethral resection of the bladder tumours followed by intravesical bacillus Calmette–Guerin therapy. After that, no recurrence of urothelial carcinoma was observed on the follow-up CT and cystoscopy during an 8-month follow-up period.

## Discussion

Ureteral pseudodiverticulosis is a relatively rare condition first reported by Holly and Sumcad in 1957.^1^ It has a predilection for involvement of the upper to middle ureter, occurs bilaterally in more than half of all cases and is more common among elderly male subjects.^2–6^ Unlike pseudodiverticulum of the alimentary tract, in which the mucosa protrudes and passes through the muscular layer because of the enhanced internal pressure, a diverticulum-like structure of the ureter is thought to form as a result of urothelial hyperplasia caused by chronic inflammation and the consequent impaction of the hyperplastic urothelium into the subepithelial connective tissue.^2–4^ Ureteral pseudodiverticulosis, including clinically unidentifiable small diverticula measuring ≤2 mm, is reportedly demonstrable in 11% of all autopsy cases.^4^ Imaging typically reveals a multifocality of tiny outpouchings in the ureter. Practically all reports pertaining to ureteral pseudodiverticulosis have mentioned the use of retrograde urography and excretory urography,^2–7^ with the rate of lesion detection being higher for retrograde urography than for excretory urography because of the higher intraureteral contrast medium pressure of retrograde urography.^3, 5,6^

CT urography has replaced excretory urography as the first-line imaging test for investigating patients with high risk for upper tract urothelial carcinoma,^8–10^ but the detection of ureteral pseudodiverticulosis using CT urography has not been previously reported. In the present case, minute outpouchings of contrast medium were noted in the right ureter during the excretory phase, whereas no such changes were identifiable during the nephrographic phase. Hydronephrosis caused by the right ureteral tumours was not observed during the nephrographic phase, while mild hydronephrosis was evident during the excretory phase. Meanwhile, our CT urography protocol requires that the bladder be filled with contrast medium during the excretory phase to improve the detection of vesical tumours; the patient is then required to void his or her bladder after completion of the nephrographic phase scanning, followed 40 min later by the excretory phase scanning.^9^ Using this procedure, the bladder is moderately dilated with contrast medium during the excretory phase, thereby increasing the upper urinary tract pressure and allowing a clear visualization of the pseudodiverticula as well as the hydronephrosis.

Since ureteral pseudodiverticulosis results from urothelial hyperplasia caused by chronic inflammation, it is suspected to be a potential risk factor for the development of urothelial carcinoma.^2, 3,5,7^ There is also a report stating that urothelial carcinoma developed in approximately 50% of ureteral pseudodiverticulosis cases.^5^ On the other hand, the high association between ureteral pseudodiverticulosis and urothelial carcinoma may reflect a sampling bias.^2, 3^ However, more cautious observation than usual and close follow-up are recommended to determine whether urothelial carcinoma develops in patients with ureteral pseudodiverticulosis unless the association between them can be denied clearly.^3, 5^

In conclusion, we presented a case of ureteral pseudodiverticulosis and its accompanying ureteral cancer diagnosed by CT urography, which is now the first-line imaging test for investigating patients with high risk for upper tract urothelial carcinoma.

## Learning points

- It is important to recognize the characteristic findings of ureteral pseudodiverticulosis on CT urography because CT urography is now widely considered as the initial imaging modality for a survey of the upper urinary tract rather than excretory urography.
- Radiologists should pay attention to accompanying urothelial carcinoma in patients with ureteral pseudodiverticulosis because it is suspected to be a potential risk factor of urothelial carcinoma.

## Consent

Written informed consent for the case to be published (including images, case history and data) was obtained from the patient for publication of this case report, including accompanying images.

## References

[1] Holly LE, Sumcad B Diverticular ureteral changes; a report of four cases. Am J Roentgenol Radium Ther Nucl Med 1957; 78: 1053–60. 13478794

[2] Cochran ST, Waisman J, Barbaric ZL Radiographic and microscopic findings in multiple ureteral diverticula. Radiology 1980; 137: 631–6. doi: 10.1148/radiology.137.3.6777826 6777826

[3] Wasserman NF, La Pointe S, Posalaky IP Ureteral pseudodiverticulosis. Radiology 1985; 155: 561–6. doi: 10.1148/radiology.155.3.3923551 3923551

[4] Wasserman NF, Posalaky IP, Dykoski R The pathology of ureteral pseudodiverticulosis. Invest Radiol 1988; 23: 592–8. doi: 10.1097/00004424-198808000-00009 3138203

[5] Wasserman NF, Zhang G, Posalaky IP, Reddy PK Ureteral pseudodiverticula: frequent association with uroepithelial malignancy. AJR Am J Roentgenol 1991; 157: 69–72. doi: 10.2214/ajr.157.1.1904678 1904678

[6] Socher SA, Dewolf WC, Morgentaler A Ureteral pseudodiverticulosis: the case for the retrograde urogram. Urology 1996; 47: 924–7. doi: 10.1016/S0090-4295(96)00055-6 8677594

[7] Parker MD, Rebsamen S, Clark RL Multiple ureteral diverticula: a possible radiographically demonstrable risk factor in development of transitional cell carcinoma. Urol Radiol 1989; 11: 45–8. doi: 10.1007/BF02926473 2499972

[8] Jinzaki M, Matsumoto K, Kikuchi E, Sato K, Horiguchi Y, Nishiwaki Y, et al Comparison of CT urography and excretory urography in the detection and localization of urothelial carcinoma of the upper urinary tract. AJR Am J Roentgenol 2011; 196: 1102–9. doi: 10.2214/AJR.10.5249 21512076

[9] Jinzaki M, Kikuchi E, Akita H, Sugiura H, Shinmoto H, Oya M Role of computed tomography urography in the clinical evaluation of upper tract urothelial carcinoma. Int J Urol 2016; 23: 284–98. doi: 10.1111/iju.13032 26750188

[10] Rouprêt M, Babjuk M, Compérat E, Zigeuner R, Sylvester RJ, Burger M, et al European Association of urology guidelines on upper urinary tract urothelial cell carcinoma: 2015 update. Eur Urol 2015; 68: 868–79. doi: 10.1016/j.eururo.2015.06.044 26188393

